# Parathyroid hormone assay: problems and opportunities

**DOI:** 10.1007/s00467-007-0508-0

**Published:** 2007-10-01

**Authors:** Kevin J. Martin, Esther A. González

**Affiliations:** Division of Nephrology, Saint Louis University, 3635 Vista Avenue, St. Louis, MO 63110 USA

**Keywords:** Parathyroid hormone, PTH, Assay, Renal osteodystrophy, Chronic kidney disease

## Abstract

The assay of parathyroid hormone continues to remain problematic as a result of the presence in the circulation of a variety of parathyroid hormone (PTH) peptides derived from secretion and from peripheral metabolism. The detection of these PTH fragments to varying degrees leads to widely differing results in the various assays used, particularly in the setting of chronic kidney disease, where PTH fragments accumulate as glomerular filtration rate (GFR) falls. The differing results not only lead to problems in comparing values from various laboratories but also limit efforts to develop useful clinical practice guidelines. At the same time, research into the precise identification of the PTH fragments which contribute to the assay problems has uncovered a relatively new area of parathyroid research that has pointed to potential biologic activity of PTH peptides previously thought to be biologically inactive and which may act on a novel PTH receptor. These issues have brought new focus to the difficulties in standardization of PTH assays and have provoked efforts to provide standards to help in the characterization of PTH assays and to facilitate the development of clinical practice guidelines.

Assay of parathyroid hormone is extremely important in clinical medicine because of the major role of parathyroid hormone in the regulation of mineral metabolism and skeletal physiology. Since the introduction of radioimmunoassay for parathyroid hormone (PTH) in 1963 by Berson et al., the assay of parathyroid hormone has been problematic and remains so to the present day [1]. The initial assays used polyclonal antisera, raised against intact parathyroid hormone, an 84 amino-acid peptide, which had been purified from parathyroid glands. Assay specificity varied considerably with different antisera and gave rise to terms such as N-terminal assays, midregion assays, and C-terminal PTH assays, according to the predominant specificity of the antisera used. While all of these assays had some clinical utility, they also allowed the characterization of the secretion and metabolism of PTH which revealed the basis for the widely different results that were obtained with different assays [2]. Thus, it was demonstrated that circulating immunoreactive parathyroid hormone was a mixture of the intact 84 amino-acid hormone peptide and several smaller molecular weight peptide fragments that were derived by secretion from the parathyroid gland as well as generated by peripheral metabolism of PTH [3–6]. The PTH peptides reacted in various assays to various degrees. Thus, the assay of parathyroid hormone was not only complicated by the reaction of these various PTH fragments in the particular assay used, but was also complicated by the fact that many of these fragments depended upon the kidney for their removal from the circulation and, accordingly, accumulated to a high degree in the setting of kidney disease [7]. While structure–function studies of parathyroid hormone indicated that the structural determinants for biologic activity resided within the first 34 amino acids, assays directed towards this region of PTH proved to be of limited value in clinical situations. Paradoxically, it appeared that radioimmunoassays with determinants towards the middle and C-terminal region of the PTH molecule appeared to provide the best clinical utility [2].

A major breakthrough in PTH assay occurred with the application of immunometric assay technology to the measurement of PTH [8, 9]. With these PTH assays, PTH was captured from serum using an antibody directed towards one region of the PTH molecule (usually towards the C-terminal region) and then detected with an antibody directed towards the other end of the PTH molecule (usually within the N-terminal 34 amino acids). Such two-site assays were believed to measure intact PTH (thus, were called “intact” PTH assays) and became widely utilized clinically and are the main type of PTH assay used at the present time. Detailed investigation over the past several years has revealed, however, that such assays are not specific for intact PTH but also detect a family of PTH peptides that are truncated at the N-terminus, a major one of which is PTH (7–84) [10, 11], as illustrated in Fig. 1. These findings not only brought a new area of confusion to the field of parathyroid hormone assay but also sparked further research, which has revealed interesting and important findings.

**Fig. 1:**
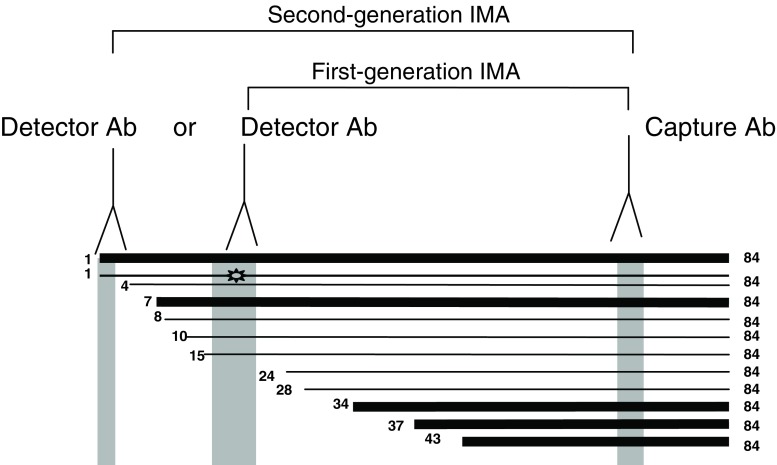
Diagram of the multiple species of PTH peptides in the circulation. The major forms are depicted by *heavy lines*. The *gray areas* depict the regions of the PTH sequence that are detected by various antibodies for first-generation and second-generation immunometric assays and indicate the PTH peptides that would be detected in each assay. The symbol () depicts a PTH 1–84 peptide that is likely post-translationally modified in a region that interferes with its detection by first-generation immunometric assays

Further refinements in assay technique, by utilizing detection antibodies that had specificity for the first four amino-acids of PTH, allowed the measurement of true PTH (1–84) and facilitated detailed investigations of parathyroid hormone assay and parathyroid hormone physiology [12]. Although such specific PTH (1–84) assays are not in widespread clinical use today, they provided a tool for detailed research on parathyroid physiology and parathyroid hormone assay. These findings led to a reinvestigation for potential biologic activity in PTH fragments that were truncated at the N-terminus of the PTH (1–84) molecule. By the use of synthetic human PTH (7–84), it was demonstrated that some biological activity could be attributed to such PTH fragments that differed in direction from that of intact PTH (1–84) [13, 14]. Thus, while intact parathyroid hormone administration elevated serum calcium, N-terminally truncated PTH fragments such as human PTH 7–84, administered to rats, appeared to lower serum calcium, and when co-administered with human PTH (1–84), blunted the calcemic response. Those observations brought new credence to the work of Murray and others from many years before, which had originally suggested that some biologic activity might be attributable to fragments of parathyroid hormone [15].

These observations have expanded our knowledge of parathyroid physiology, but, at the same time, they have focused attention on some significant clinical problems. One such problem is that even with current immunometric assay techniques, there is considerable heterogeneity in results obtained using different assays from different manufacturers [16]. The widely different results obtained likely stems from the variable recognition of PTH fragments such as PTH (7–84) by the various assays used. An additional problem which follows is the questions that surround the development and application of practice guidelines for appropriate therapeutic targets for the desirable levels of parathyroid hormone with regard to the control of hyperparathyroidism in the setting of chronic kidney disease. Thus, a pressing clinical issue is how to compare results from one laboratory with those of another, with regard to the development of practice guidelines.

On the other hand, these apparent problems in PTH assay also lead to a number of opportunities that may help the field move forward. First, the recognition of potential biologic activity for N-terminally truncated PTH fragments provides a stimulus to try and uncover and define the physiological importance of the biological role for such PTH fragments in humans. Some evidence in vitro and in animal experiments has been developed to indicate the likely presence of a specific receptor for such PTH fragments (C-PTH-R), and preliminary data have suggested that such receptors might exist particularly in cells of the osteocyte lineage, thereby opening a new field of parathyroid biology [17–19]. These observations also provide the opportunity to learn how such PTH fragments might function in a milieu where many types of PTH fragments exist and circulate, such as in chronic kidney disease, and, thus, the opportunity exists to learn whether these PTH fragments will have a spectrum of activities when they exist alone or in combination. The C-PTH-R differs from the PTH receptor (PTH-R1), which is responsible for the classical actions of PTH. PTH fragments with a truncated N-terminus, such as PTH 7–84, do not bind to the PTH-R1 [20].

An additional problem raised by these observations is the difficulty in trying to standardize PTH assay results from different laboratories or reagent suppliers using the various techniques and to achieve precise characterization of antisera used. This issue is not unique to the assay of PTH but is also relevant to other peptide hormone assays, such as the assay of growth hormone [21]. The provision of accurate PTH standards that could be used worldwide would be helpful in this regard. Similarly, interpretation of results obtained at the present time might also be facilitated by the provision of biological samples on a worldwide basis that could aid in the general characterization of assays used and help with clinical decision making, particularly in the setting of chronic kidney disease. These observations also provide the stimulus and opportunity to revisit the use of parathyroid hormone assay as a bone marker for the management of renal osteodystrophy, and will likely lead to a new era of bone biopsy to define correlations of parathyroid hormone values and other biomarkers in the current therapeutic era to try and facilitate clinical management. To this point in time, intact PTH measurements have been a relatively imprecise predictor of bone turnover [22–24]. It has also been suggested that measurement of N-terminally truncated PTH fragments such as PTH (7–84), together with the values for PTH (1–84), might provide useful information [25, 26]. This is an extremely controversial area that requires much additional work to define the biological role for PTH (7–84) in humans in a complex milieu of other PTH fragments and to understand the factors that may alter the relative amounts of PTH and fragments, before clinical decision making should be based upon such measurements [27–32].

In summary, problems with the assay of parathyroid hormone continue but have uncovered new areas of parathyroid hormone physiology and opened a new area of investigation into potential biologic activity of PTH fragments. As these assay problems are solved, and efforts at standardization of PTH assays continue, it is likely that we will be able to understand the total spectrum of biologic activity of PTH and PTH fragments and incorporate the information into clinical practice to improve the assessment and management of abnormalities of mineral metabolism in a variety of clinical circumstances.
